# Recent Progress in Flexible Microelectrode Arrays for Combined Electrophysiological and Electrochemical Sensing

**DOI:** 10.3390/bios15020100

**Published:** 2025-02-10

**Authors:** Umisha Siwakoti, Steven A. Jones, Deepak Kumbhare, Xinyan Tracy Cui, Elisa Castagnola

**Affiliations:** 1 Department of Biomedical Engineering, Louisiana Tech University, Ruston, LA 71272, USA; usi002@latech.edu (U.S.); sajones@coes.latech.edu (S.A.J.); 2 Department of Neurosurgery, Louisiana State University Health Sciences, Shreveport, LA 71103, USA; deepak.kumbhare@lsuhs.edu; 3 Department of Bioengineering, University of Pittsburg, Pittsburgh, PA 15260, USA; xic11@pitt.edu; 4 Center for Neural Basis of Cognition, University of Pittsburgh, Pittsburgh, PA 15213, USA; 5 McGowan Institute for Regenerative Medicine, University of Pittsburgh, Pittsburgh, PA 15219, USA; 6 Institute for Micromanufacturing, Louisiana Tech University, Ruston, LA 71272, USA

**Keywords:** flexible MEAs, neurotransmitter detection, electrophysiology

## Abstract

Understanding brain function requires advanced neural probes to monitor electrical and chemical signaling across multiple timescales and brain regions. Microelectrode arrays (MEAs) are widely used to record neurophysiological activity across various depths and brain regions, providing single-unit resolution for extended periods. Recent advancements in flexible MEAs, built on micrometer-thick polymer substrates, have improved integration with brain tissue by mimicking the brain’s soft nature, reducing mechanical trauma and inflammation. These flexible, subcellular-scale MEAs can record stable neural signals for months, making them ideal for long-term studies. In addition to electrical recording, MEAs have been functionalized for electrochemical neurotransmitter detection. Electroactive neurotransmitters, such as dopamine, serotonin, and adenosine, can be directly measured via electrochemical methods, particularly on carbon-based surfaces. For non-electroactive neurotransmitters like acetylcholine, glutamate, and γ-aminobutyric acid, alternative strategies, such as enzyme immobilization and aptamer-based recognition, are employed to generate electrochemical signals. This review highlights recent developments in flexible MEA fabrication and functionalization to achieve both electrochemical and electrophysiological recordings, minimizing sensor fowling and brain damage when implanted long-term. It covers multi-time scale neurotransmitter detection, development of conducting polymer and nanomaterial composite coatings to enhance sensitivity, incorporation of enzyme and aptamer-based recognition methods, and the integration of carbon electrodes on flexible MEAs. Finally, it summarizes strategies to acquire electrochemical and electrophysiological measurements from the same device.

## 1. Introduction

Development of a complete understanding of brain function remains one of the greatest challenges for both neuroscientists and neural engineers.

Neural communication involves both chemical and electrical signaling, with neurotransmitters operating on different timescales and interacting to regulate specific brain functions. Advancing our understanding of these intricate communication mechanisms requires multimodal implantable neural probes capable of concurrently monitoring electrophysiological signals and the multi-time-scale chemical dynamics (tonic and phasic) of multiple neurotransmitters across multiple brain locations, over extended time periods. By elucidating the interplay between electrical and chemical signaling, the role of these signals in the pathogenesis of neurological and psychiatric disorders, and the mechanisms that underpin the efficacy of electrical stimulation for neurotransmission and neuromodulation [1,2,3], these devices have the potential to revolutionize our understanding of fundamental neuroscience principles. This foundational knowledge is critical to enable targeted therapeutic interventions for a range of neurological and psychiatric disorders, such as Parkinson’s disease, depression, and epilepsy [4,5,6]. For example, chronic multimodal monitoring is essential for supporting closed-loop neuromodulation systems, where continuous recording of the neural environment is required to deliver appropriate stimuli, thereby achieving precise closed-loop control [6,7,8]. These systems include adaptive deep brain stimulation, used to treat Parkinson’s disease (PD) and other motor disorders [8,9,10], as well as closed-loop systems designed to detect signal anomalies in epileptic patients and deliver stimuli to suppress seizures [6,11].

Microelectrode arrays (MEAs) are widely used to record neurophysiological activity with single-neuron resolution across varying brain depths and widths, maintaining functionality over weeks to years [12]. However, stiff silicon-based MEA implants create a “kill zone”, characterized by reduced neuron density and increased glial encapsulation [13,14,15], which impairs electrophysiological and neurochemical measurements during chronic use [16,17,18]. Implant instability is attributed to several factors, including the foreign body response, neural degeneration, material degradation, and mechanical mismatch between the brain tissue and the implant [19,20]. For instance, probe penetration into neural tissue creates acute damage, leading to the destruction of cells, extracellular matrices, and capillaries, and to the disruption of the blood–brain barrier (BBB) [21]. While some degree of recovery of the initial damage may occur [22], repeated micro-motions of the implanted device relative to the surrounding tissue aggravate inflammatory responses [21,23]. This effect is exacerbated by the mechanical mismatch between the soft, low-modulus neural tissue (with elasticity in the range of a few kPa) and the much stiffer materials used in implantable probes (which can reach hundreds of GPa for metals and silicon). This reactive tissue response leads to the subsequent formation of a high impedance encapsulation sheath, known as a glial scar [23]. The presence of the scar tissue around the electrode impairs both signal recording and neural stimulation, displacing surrounding neurons and electrically insulating the electrode from the neighboring brain regions [24,25].

Additionally, conventional MEAs typically incorporate metal microelectrodes (Au, Pt, Ir) that present poor sensitivity towards electroactive neurotransmitters, such as dopamine (DA), serotonin or 5-hydroxytryptamine (5-HT), and adenosine (AD).

In the past decade, significant efforts have been directed to tune the structural, functional, and dimensional properties of conductive and insulating components of these devices, with the aim of achieving appropriate chemical and electrical properties and matching the mechanical properties to those of the nervous system. Notably, micrometer-thick polymers, such as polyimide, parylene C, and SU-8, have been used as substrates for flexible MEAs to mimic the mechanical properties of soft brain tissue, thereby minimizing perpetual mechanical trauma and inflammation [26,27,28]. These flexible MEAs, along with subcellular scale features, have demonstrated seamless integration with neural tissue for stable, long-term recordings [27,28,29,30,31,32]. Furthermore, achieving a high signal-to-noise-ratio, long-term electrical performance, and stable electrochemical detection necessitates the careful selection of conductive electrode materials. Various material coatings, such as iridium oxide [33,34,35,36], nano platinum [37,38], and conductive polymers [39,40,41,42], have been employed to reduce impedance and enhance the charge-transfer capability of metal-based MEAs. More recently, custom functionalization of flexible MEAs has enabled the electrochemical detection of both *electroactive* and *non-electroactive* neurotransmitters [39,43,44].

The inherent redox properties of electroactive neurotransmitters, including DA, 5-HT, AD, and melatonin (MT), allow them to be directly measured with electrochemical methods, preferentially at carbon surfaces [45,46,47,48,49]. In contrast, non-electroactive neurotransmitters, including acetylcholine (ACh), glutamate (GLU), and γ-aminobutyric acid (GABA), require alternative strategies for electrochemical detection [50,51]. These strategies include immobilization of target-specific enzymes on the microelectrode surface to catalyze reactions that generate electroactive byproducts, such as hydrogen peroxide (H_2_O_2_), which can then be detected electrochemically [51,52]. Additionally, aptamers—oligomers of artificial ssDNA, RNA, Xeno nucleic acid (XNA), or peptide designed to bind specifically to target molecules—can be immobilized on the microelectrode surface and engineered to undergo conformational changes upon target binding, generating electrochemical signals [44,53,54,55,56].

This review summarizes recent advancements in flexible MEA fabrication and functionalization to achieve the following: (1) multi-time scale (tonic and phasic) neurotransmitter detection, (2) flexible MEA functionalization strategies to combine electrophysiological and electrochemical sensing, including (a) composite coatings, and (b) incorporation of enzyme and aptamer, (3) integration of carbon electrodes on flexible MEAs, and (4) acquisition of electrochemical and electrophysiological measurements from a single device.

## 2. Tonic and Phasic Electrochemical Detection of Neurotransmitters Using Flexible MEAs

Neurotransmitter release occurs over multiple timescales, including tonic and phasic release. Tonic release reflects the slower, continuous firing of neurons, which maintains the extracellular basal levels through extra synaptic diffusion (seconds to minutes [57,58]). Phasic release results from rapid burst-firing of neurons, leading to fast, high concentration bursts in the synaptic cleft (milliseconds to seconds), caused by neuronal firing in response to stimuli [57,59].

**Figure 1:**
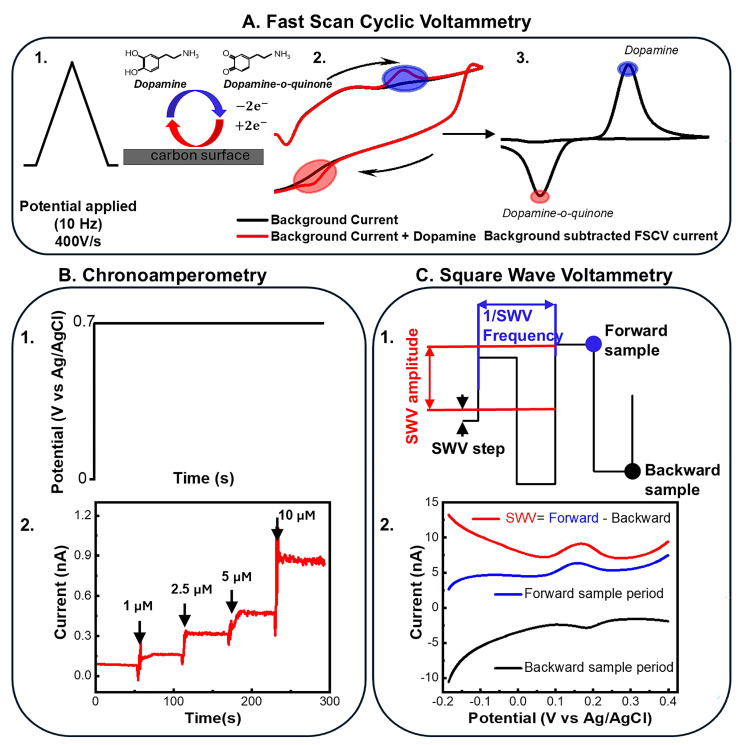
(**A**) Fast Scan Cyclic Voltammetry (FSCV); example of dopamine (DA) detection. After the application of an FSCV waveform from −0.4 to 1.3 V with scan rate of 400 V/s at 10 Hz at carbon microelectrode (1.), a background current is generated both in the presence (red) and absence (black) of 1 μM DA (2.). By subtracting the background signal, the DA oxidation and DA-o-quinone reduction peaks are isolated (3.). (**B**) Chronoamperometry; example of H_2_O_2_ detection. (1.) Waveform of amperometry at a voltage of +0.7 V. (2.) nanoPt-coated electrode’s current response to 1–10 µM of H_2_O_2_ concentrations. (**C**) Schematic of a Square Wave Voltammetry (SWV). (1.) SWV is a type of staircase voltammetry that applies a symmetric square-wave pulse superimposed on a staircase potential waveform. The current is measured at the end of each pulse, allowing for high sensitivity by effectively reducing background noise and isolating the signal from the redox reaction of interest. The forward current is recorded at the end of each anodic hold period (blue marker), while the backward current is recorded at the end of each cathodic hold period (black marker). (2.) SWV detection of a 1 μM DA concentration at a PEDOT/CNT-coated GC microelectrode. The measurement shows an oxidation peak in the forward scan (blue) and a reduction peak in the backward scan (black). The final SWV current response is the difference between the forward and backward current responses (red).

Fast Scan Cyclic Voltammetry (FSCV) at carbon fiber microelectrodes (CFEs) has been considered the gold standard for *in vivo* detection of electroactive neurotransmitters, significantly advancing our understanding of their phasic dynamics [60,61,62]. A detailed description of FSCV has been provided in previous review papers [46,48,63,64]. A schematic of a typical FSCV waveform is presented in Figure 1A. FSCV relies on the direct electron transfer reaction between redox-active molecules and the carbon surface of the electrodes [46,48,64,65]. By sweeping a potential waveform at fast scan rates (400–1200 V/s), FSCV achieves a sub-second temporal resolution [66]. Different FSCV waveforms have been investigated for different analytes, including DA, 5-HT, AD, MT, guanosine (GN), and methionine-enkephalin (M-ENK), to identify the combination of holding/switching potentials and scan rate that maximizes sensitivity and minimizes fouling [46,49,67,68,69,70,71,72,73]. The waveform is scanned at a repetition frequency of 10 Hz (100 ms temporal resolution). Between scans, a holding potential is applied to the working electrode to selectively preconcentrate the target molecule on its surface [48,64,65,73,74]. While FSCV can measure phasic electroactive neurotransmitter release (rapid changes in concentrations), the necessity for background subtraction has prevented its use for tonic measurement [46,48]. Tonic neurotransmitter dynamics play a crucial role in modulating neural activity and behavior outcomes [66,75]. Moreover, alterations in the interaction between tonic and phasic neurotransmitter dynamics lead to abnormal neurological function and are implicated in multiple neurological disorders [58,76,77,78,79,80]. Therefore, MEAs capable of measuring both phasic and tonic neurotransmitter releases across multiple brain regions are essential for understanding brain function and dysfunction, advancing the diagnosis and treatment of these disorders.

Amperometry techniques, such as chronoamperometry and constant potential amperometry (CPA), if properly self-referenced [81], can measure phasic and tonic neurochemical events with high temporal resolution, enabling millisecond-scale continuous recording for hours without significantly interfering with electrophysiological signals [81,82,83,84]. An example of chronoamperometry waveform for H_2_O_2_ detection is presented in Figure 1B. However, its primary limitation is the lack of selectivity, as multiple electroactive species can contribute to the recorded current, complicating target identification. To tune selectivity, strategies such as modifying the applied potential to a value that is more sensitive to the desired analyte but less responsive to interferents, and/or applying selective membrane coatings are employed, minimizing interference and enhancing specificity [50,51,85,86]. Functionalized MEAs have been used for multi-channel amperometry detection of multiple neurotransmitters dynamics, both electroactive and not electroactive [50,87,88,89,90]. For example, ceramic MEAs, with Nafion-coated-Pt electrodes, with or without m-phenylenediamine (m-PD), enabled tonic and phasic DA in rat striatum with CPA. This measurement was facilitated by a self-referencing amperometric method that subtracted signal from Nafion-coated electrodes and m-PD/Nafion-coated electrodes (sentinels) in real-time [83]. Nafion acted as a selective barrier against negatively charged interferents, such as ascorbic acid (AA) and 3,4-dihydroxyphenylacetic acid (DOPAC), while m-PD served as a size exclusion filter, blocking larger molecules, including DA, from accessing the Pt recording sites [83].

Alternative electrochemical methods have been recently developed and optimized for tonic DA and 5-HT detection, including fast-scan controlled-adsorption voltammetry (FSCAV) [91,92,93], charge-balancing multiple waveform FSCV [94], convolution-based FSCV [95,96], fast cyclic square-wave voltammetry (SWV) [97,98], and N-shaped multiple cyclic SWV [99]. These techniques have been successfully applied *in vivo* and demonstrated successful detection of extracellular tonic DA concentrations in a ~50–100 nM range, and 5-HT concentrations in a ~60 nM range. However, these measures have been carried out at single CFEs, limited to a single active site per penetrating electrode [11,60,91,92,93,95,96,97,98,99,100]. Because neurotransmitter dynamics are complex and differ across various brain regions and even within subareas of the same region, high-resolution multisite measurements would be preferred [101,102,103,104,105].

Recently, SWV waveforms have been optimized to detect basal DA and 5-HT levels in rodent brains, both at poly(3,4-ethylenedioxythiophene) (PEDOT)/carbon nanotubes (CNT)-coated (PEDOT/CNT) and bare glassy carbon (GC) MEAs [43,106,107]. SWV is a pulse voltammetry technique in which a symmetrical square-wave pulse is superimposed on a staircase potential waveform. The current is sampled twice during each cycle: once at the end of the forward square-wave pulse and again at the end of the reverse pulse. The resulting signal is defined as the difference between the current measured during the forward and reverse pulses. SWV presents high sensitivity and effective isolation of faradaic currents—arising from redox reactions of electroactive analytes—from capacitive charging currents, thereby enabling precise detection of basal neurotransmitter levels [43,106,107]. The tonic DA and 5-HT concentration measurements obtained with SWV are comparable to those obtained with the previously mentioned voltammetric techniques [43,106,107]. A schematic of the SWV waveform is presented in Figure 1C.

Recently, the first implantable flexible MEA capable of multisite electrochemical sensing of both tonic and phasic DA dynamics *in vivo* has been demonstrated [43]. Phasic DA detection was obtained with FSCV at GC microelectrodes. Tonic DA detection was measured using an optimized SWV waveform in combination with PEDOT/CNT-coated microelectrodes [43]. More recently, miniaturized GC fiber-like (GCF) MEAs have been successfully used to measure tonic DA and 5-HT concentrations *in vivo* using SWV and stimulation-evoked phasic DA via FSCV. These devices also recorded neural activity in the striatum of mouse brains [106]. This novel design holds significant potential for multimodal measurements of neural activity and neurotransmitter concentrations, while maintaining an exceptionally minimal footprint (see Section 3).

## 3. Conductive Polymers and Composite Coatings for Electrophysiological Recording and Neurotransmitter Detection

Conductive polymers, such as polypyrrole (PPy) and poly(3,4-ethylenedioxythiophene) (PEDOT), have gained considerable attention as neural electrode coatings due to their superior electrochemical performance compared to metal electrodes [108,109,110,111,112,113,114]. These polymers, characterized by a conjugated backbone, achieve conductivity through oxidation (doping), which introduces charge carriers while maintaining charge neutrality with counter ions. Among various conducting polymers, PEDOT has shown superior chemical and electrochemical stability, high conductivity, and mechanical durability. In PEDOT, the oxygen bridge between the 3- and 4-positions of the thiophene ring prevents α–β coupling, preserving the polymer’s structure and ensuring a more stable conjugated backbone. This structural stability translates into enhanced electrochemical durability, making PEDOT highly resistant to degradation in biological environments. As a result, PEDOT is particularly well-suited for long-term *in vivo* neural applications, including biosensors, bioelectronics, and brain–machine interfaces (BMI) [40,115].

The high surface area and ionic conductivity of PEDOT-based coatings drastically decrease the electrochemical impedance of metal electrodes, enabling high signal-to-noise ratio (SNR) recordings and enhanced charge injection capacity [40,41,116]. Thanks to its proven ability to improve recording performance, PEDOT:PSS has been used as a standard electrode coating for some of the most advanced flexible neural electrode designs, including the NeuroGrid [41], Neuralink [117], and NET devices [28].

Despite their superior electrochemical properties, such as low impedance and high charge-transfer capability, when subjected to prolonged electrical stimulation during *in vivo* use, PEDOT-based coatings resulted in decreased charge injection limit, increased impedance, and delamination from the underlying substrate [36,118]. Different strategies have been investigated to improve PEDOT adhesion and long-term stability, with varying degrees of success. These strategies include substrate modification and functionalization [108,111,119,120,121], and the use of different dopants or co-dopants [122,123]. A comprehensive overview of recent developments in utilizing PEDOT and various counterions (dopants) for stimulating electrodes has been summarized and discussed in our previous review by Zheng et al. [119].

In this review, we primarily focus on the application of PEDOT-based coatings for sensing, with particular emphasis on their critical role in enabling simultaneous electrophysiological and electrochemical recordings using flexible MEAs *in vivo.*

In addition to their benefits for electrophysiology, PEDOT-based composite coatings have been extensively investigated for neurotransmitter detection [107,124,125,126,127].

PEDOT:Nafion has been used to improve CFE sensitivity and fouling resistance in FSCV measurements. Electropolymerized PEDOT:Nafion significantly enhanced detection sensitivity for DA [126,127,128], 5-HT [128], and AD [128] compared to bare CFEs. Additionally, these coatings substantially reduced acute *in vivo* biofouling [126].

The incorporation of nanocarbon materials into PEDOT coatings, such as two-dimensional graphene oxide (GO) sheets [125] and acid-functionalized CNTs [43,107,124], has shown particular promise, not only in improving electrical stability and conductivity but also facilitating the absorption of monoamine neurotransmitters, thanks to the presence of functional groups. For example, PEDOT/graphene oxide (GO)-modified (PEDOT/GO) CFEs have been developed for enhanced DA detection using FSCV in rat dorsal striatum [125]. PEDOT/GO coatings have demonstrated an 880% increase in sensitivity and a 50% decrease in the limit of detection for DA compared to bare CFEs [125]. However, the thickness of these coatings must be carefully tuned to balance the increase in sensitivity without compromising adsorption or electron transfer kinetics. The enhanced DA sensitivity is attributed to the increased effective surface area and improved adsorption of DA’s oxidation product (DA-o-quinone) [125].

More recently, highly conductive PEDOT/CNT composite coatings have been incorporated into CFEs and optimized for highly sensitive and selective detection of resting DA levels in the rat striatum using SWV [124]. This electrochemical technique allows for the direct measurement of tonic DA levels by selectively eliminating non-faradaic background charging currents. PEDOT/CNT functionalization significantly enhanced electrode sensitivity for SWV-based DA detection, increasing sensitivity by 422-fold compared to bare CFEs [124]. Notably, when selectively electropolymerized onto the metal recording site of silicon MEAs, these coatings enabled the first time-correlated, multisite quantification of basal DA levels across different layers of the rat brain [124]. Similarly PEDOT/CNT-coated microelectrodes previously demonstrated improved neural recording stability over four months compared to PEDOT:PSS recording sites [129].

While PEDOT-based coatings, particularly those incorporating nanocarbon, effectively reduce impedance for electrophysiological recordings, they are also crucial for enabling electrochemical neurotransmitter detection. When coated onto metal MEAs, these coatings allow direct electrochemical sensing of neurotransmitters, which would otherwise be impossible with unmodified metal electrodes due to their poor sensitivity to electroactive neurotransmitters. Building on PEDOT’s dual functionality, researchers have integrated PEDOT composite coatings into both stiff [130,131] and flexible [39,132,133] MEAs to enable dual measurement of both neurotransmitter concentrations and neural activity. While electrophysiological recordings capture neural activity, including extracellular action potentials and local field potentials, electrochemical sensing detects neurotransmitters in real-time, providing complementary insights into brain function. PEDOT-coated flexible MEAs integrate these modalities, linking electrical and chemical signaling for a more comprehensive neural analysis. This multimodal approach is crucial for deepening our understanding of brain function in both healthy and diseased states.

PEDOT/CNT coatings have recently been applied to flexible GC-MEAs [43,107]. When applied to bare GC microelectrodes, PEDOT/CNT increased DA sensitivity approximately sixfold using the previously optimized DA SWV waveform [43]. In a separate study, PEDOT/CNT-coated GC microelectrodes demonstrated 16 times higher sensitivity towards 5-HT than CFEs, even though the geometric area of the CFEs was seven times larger [107]. This enhanced sensitivity demonstrates the effectiveness of the optimized SWV waveform at PEDOT/CNT for detecting tonic 5-HT concentrations. Using this SWV waveform optimized for 5-HT, PEDOT/CNT-coated GC microelectrodes also show high 5-HT selectivity and fouling resistance [107]. The high sensitivity of the PEDOT/CNT-coated GC microelectrodes can be attributed to a much larger effective surface area of the PEDOT/CNT nanocomposite, as evidenced by reduced electrochemical impedance and increased charge-transfer capabilities [43,107]. Furthermore, the incorporation of negatively charged functional groups on the surface of the electrode facilitates DA and 5-HT adsorption through electrostatic interactions, which have been shown to enhance their detection. Therefore, both the increased negative charge and the larger surface area contribute to enhanced sensitivity. These PEDOT/CNT-coated flexible GC-MEAs have enabled stable multisite *in vivo* detection of tonic DA for 21 days [43] and 5-HT for at least 7 days [107]. The chronic sensing stability may be attributed to several factors, including the high stability of the PEDOT/CNT coating on GC [43,107,134], the excellent electrochemical stability of both PEDOT/CNT [122,134] and GC [135,136], the high fouling resistance of negatively charged acid-functionalized CNTs [43,107,137,138], and the minimal glial inflammatory response induced by the flexible substrate [43,107].

A single-wall carbon nanotube (SWCNTs)/PEDOT:PSS nanocomposite coating was applied to a 16 channel four-shank implantable silicon-based MEA to significantly enhance its electrical and electrochemical performance [130]. The improved electrical properties enable the MEA to detect electrophysiological signals with a high signal-to-noise ratio (SNR), while the enhanced electrochemical properties allow the MEA to respond sensitively and selectively to DA concentrations, using chronoamperometry at low oxidation potential (160 mV). The CNTs/PEDOT:PSS-modified MEA was used to acutely detect dual-mode signals, including electrophysiological activity and DA concentration, in the rat striatum under isoflurane anesthesia [130].

A composite of platinum black nanoparticles and PEDOT:PSS (PtNPs/PEDOT:PSS) was electrodeposited onto parylene C-based flexible MEAs to simultaneously measure spike firing and DA concentration [132]. DA detection was achieved using chronoamperometry at an oxidation potential of 0.35 V, with selectivity enhanced through the application of Nafion coating. These dual-mode flexible MEAs were utilized to monitor electrophysiological activity and dynamic changes in DA levels in the striatum of awake mice under fear-inducing conditions. The results revealed that after exposure to fear stimuli, decreases in the mean behavioral activity of the mice were accompanied by synchronous reductions in action potential firing rates, low-frequency (0–30 Hz) local field potential (LFP) power, and DA concentrations in the striatum. This study demonstrated the practicality of these flexible MEAs for the simultaneous detection of electrophysiology and DA dynamics [132].

**Figure 2:**
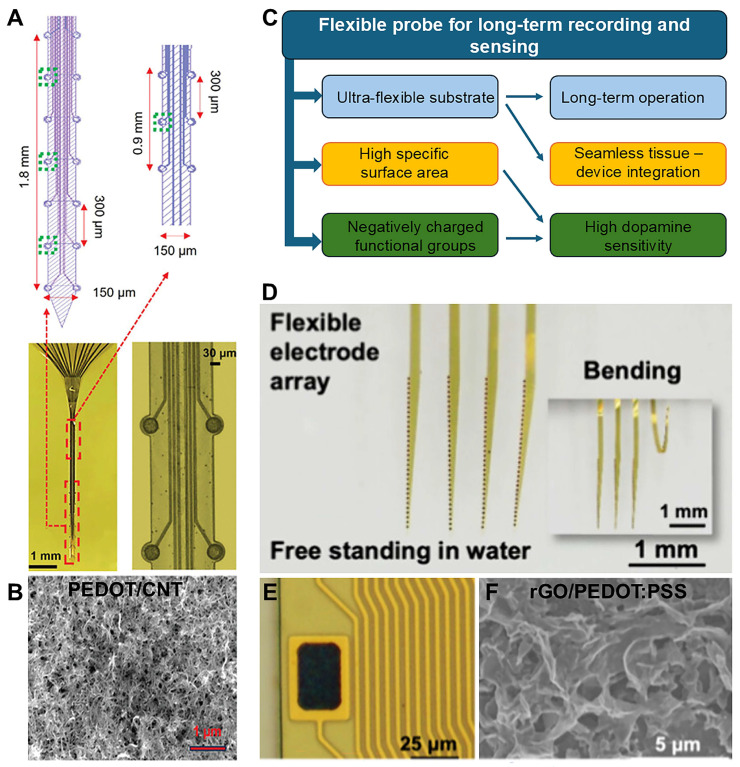
(**A**) MEA design layout (**top**) and optical images of the fabricated MEAs (**bottom**). The shank measures 150 µm in width, 12 µm in thickness, and 5 mm in length, with a 300 µm spacing between individual sites. The MEA consists of 20 channels, with only the 4 sites highlighted in green used for DA sensing. The optical image of the MEA shank shows two regions: Region 1 (striatum) contains 14 sites, while Region 2 (cortex) has 6 sites. Each site has a diameter of 35 µm. Adapted from Wu et al. [39]. (**B**) SEM image of PEDOT/CNT coating on top of Pt recording sites. Adapted from Wu et al. [39]. Copyright © 1999–2025 John Wiley & Sons, Inc. This publication is licensed under CC-BY 4.0. (**C**) Advantages of electrical and electrochemical performance of flexible rGO/PEDOT:PSS and PEDOT/CNT-modified electrodes. Adapted with permission from Wang et al. [133]. (**D**) Optical image of released flexible electrode arrays (MEAs) in water. (**E**) Optical microscopic image of the modified electrode sites. Adapted with permission from Wang et al. [133]. (**F**) SEM image of rGO/PEDOT:PSS coating. Adapted with permission from Wang et al. [133]. Copyright © 2024 American Chemical Society.

PEDOT/CNT coatings were deposited on microfabricated flexible 20-channel polyimide MEAs for SWV-based dual-mode longitudinal detection of electrophysiological signals and tonic DA concentrations using SWV (Figure 2A,B). Similarly prepared PEDOT/CNT coatings have previously demonstrated significantly enhanced electrode sensitivity for SWV-based DA and 5-HT detection compared to bare CFEs and GC-MEA microelectrodes [124], with high selectivity and fouling resistance [107]. The enhanced sensitivity of PEDOT/CNT-coated MEAs has been attributed to the larger effective surface area of the PEDOT/CNT. Additionally, the negatively charged functional groups on the acid-functionalized CNTs facilitate DA and 5-HT adsorption, further improving detection sensitivity [39,43,107].

The customized MEA channel layout (Figure 2A) also enabled simultaneous monitoring of both cortical and striatal regions. These MEAs were adopted to investigate the involvement of DA dynamics in circadian rhythm regulation [39]. They were implanted in wild-type (WT) and Δ19 *Clock* mutant (MU) mice, which were hypothesized to exhibit changes in dopaminergic transmission, and recorded tonic DA concentrations and extracellular neural activity with high spatial and temporal resolution over four weeks. A diurnal fluctuation in DA concentration was observed in WT mice, but not in MU mice, which also exhibited higher basal DA concentrations and a stronger response to cocaine [39]. Additionally, the striatal neuronal firing rate was found to be positively correlated with DA concentration in both animal groups [39]. These measurements revealed that extracellular DA in the striatum is affected by the *Clock* gene.

Reduced graphene oxide (rGO)-doped PEDOT:PSS (rGO/PEDOT:PSS) coatings were electropolymerized into 128-channel ultrathin polyimide flexible MEAs to enhance DA sensitivity and electrical performance, enabling synchronous detection of electrical and electrochemical signals [133] (Figure 2C–F). Similar to PEDOT/CNT, rGO/PEDOT:PSS coatings provided a large effective surface area rich in negatively charged functional groups, enhancing DA sensitivity [133]. DA levels were measured via constant potential amperometry at 0.2 V vs. Ag/AgCl, ensuring stable voltage throughout the measurement with only brief crosstalk at the start and end of the recording. Given the presence of other electrochemically active substances in the body that can be oxidized at the same potential as DA, the electrode surface was coated with Nafion coating to enhance selectivity. These ultra-flexible devices (2.5 µm thick), with four shanks of 32 uniformly distributed microelectrodes, were implanted into the caudate putamen of the mouse brain, crossing multiple regions, including the striatum, motor/primary sensory cortex, and deep brain regions simultaneously. The rGO/PEDOT:PSS-modified flexible MEAs reliably recorded local field potentials (LFPs), single-neuron activity, and DA fluctuations for over six weeks *in vivo*, while ensuring biocompatibility. These MEAs were used to observe and analyze the effects of nomifensine on both electrical and electrochemical brain activity, offering valuable insights into its *in vivo* mechanism of action and revealing the interplay and synergy between DA signaling and neural activity in different brain regions. Additionally, monitoring changes in the mice’s trajectories allowed for the correlation of behavioral alterations with corresponding changes in brain activity [133].

Table 1 summarizes the most relevant results achieved using MEAs functionalized with PEDOT nanocomposite coatings for sensing, particularly its role in enabling concurrent electrophysiological and electrochemical sensing using flexible MEAs. Notably, the most significant results in chronic dual-mode detection has been achieved using flexible MEAs functionalized with PEDOT doped with nanocarbon materials, i.e., PEDOT/CNT [39] and rGO/PEDOT:PSS [133].

The chronic sensing stability of these flexible MEAs [40,134] may be attributed to several factors, including the good electrochemical stability of rGO/PEDOT:PSS [133] and PEDOT/CNT [41,124,136], the high fouling resistance of negatively charged acid-functionalized nanocarbon materials [39,43,107,137,138], and the minimal glial inflammatory response induced by the flexible substrate [39,43,107] (Figure 2C).

The dual measurement capability and chronic reliability of these flexible MEAs coated with conductive polymer–nanocarbon composites highlight their potential for long-term *in vivo* electrochemical and electrophysiological monitoring, thus offering a powerful tool for advancing the understanding of brain function and driving breakthroughs in a wide range of neuroscience research areas (Figure 2C).

## 4. Enzymatic and Aptamer-Based Detection

**Figure 3:**
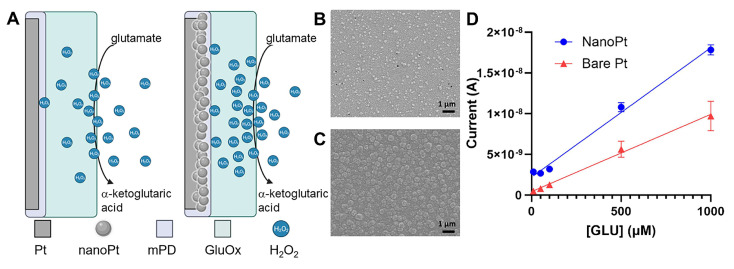
(**A**) A schematic illustration depicting the enzymatic detection mechanism of GLU using Pt and nanoPt microelectrode. (**B**,**C**) Scanning electron microscope (SEM) images showing smooth Pt (**B**) and porous NanoPt (**C**). (**D**) Calibration curves comparing the GLU sensitivity of nanoPt microelectrodes (blue) with that of smooth Pt microelectrodes (red). Adapted from [50]. Copyright © 2024 The Authors. Published by American Chemical Society. This publication is licensed under CC-BY 4.0.

Another type of electrochemical detection utilizes the immobilization of biological recognition elements onto the microelectrode’s surface [50,51,139]. Typical recognition elements include target-specific enzymes or aptamers that interact with the target NTs to generate a measurable electrochemical response, enabling sensitive detection [44,50,51,86,87,88,89,140,141,142,143,144]. NT-specific enzymes immobilized on the microelectrode sites convert non-electroactive NTs into electroactive byproducts, such as hydrogen peroxide (H_2_O_2_), which can be detected using standard electrochemical methods, usually chronoamperometry [50,51,87,88,89,140,141,142,143,144]. NT-specific enzymes have also been used to detect electroactive NTs from metal microelectrodes [83,145,146]. A representative schematic representation of the mechanism of the Pt and nanoPt GLU sensor is provided in Figure 3A.

Electrochemical detection of ACh, GLU, and GABA from ceramic MEAs has been established [81,83,87,89,140,141,147]. The MEA Pt electrodes have also been functionalized for multianalyte detection on the same device, including GLU and GABA [50,52,141], GLU and ACh [89], DA and GLU [90,131], choline (Ch) and ACh [88], Ch, GLU, and glucose and lactate [148]. Pt electrodes are often preferred for their desirable catalytic effect on the oxidation of H_2_O_2_. An enzyme/crosslinker layer is typically deposited on the single recording sites of the array by manual drop casting. Size-exclusion screening layers are applied to reduce the influences of interfering molecules [50,51,52]. An enzyme-free ‘sentinel’ electrode on the array is used as a reference to subtract the background H_2_O_2_ signal.

Silicon-based MEA technology has also been used to simultaneously detect neurotransmitters and electrophysiological signals *in vivo* [131,148,149,150]. Manual drop casting can limit the throughput and requires a large electrode size (15 × 333 µm [87,89] 50 × 150 µm [147]) not compatible with single-unit recordings. Crosslinking may also negatively affect the electrode impedance for electrophysiology measurements. High-surface-area coating, such as platinum nanoparticles (PtNPs) and reduced graphene oxide nanocomposites (Pt/rGOs), have been used to lower the impedance of recording sites and increase the sensitivity of sensing sites, additionally functionalized with an enzyme/crosslinker layer [131,149]. MEAs modified with these coatings demonstrated the ability to dynamically record changes in neurotransmitters and neurophysiology activity in the hippocampus sub-regions of epileptic anesthetized rats [149], and awake mice [131]. Different electrodes of the same MEA were dedicated to electrical recording or chronoamperometry neurotransmitter detection, but the dual-mode recordings were obtained simultaneously. These MEAs revealed that seizures in epileptic mice strongly correlate with changes in GLU, DA, and neuronal activity, which occurred before visible behavioral symptoms. The synchronized signal changes in the pre-seizure phase suggest the potential for early seizure prediction. This technology offers a valuable tool for studying epilepsy mechanisms [131].

However, chronic sensing over days and weeks *in vivo* has been challenging, possibly due to enzyme instability and/or foreign body reactions [151,152,153]. While flexible substrates can seamlessly integrate with the neural tissue [27,28,107], porous electrode materials are highly beneficial for enzyme immobilization [154,155,156]. Porous structures increase the effective surface area, enabling higher enzyme anchoring, minimizing the risk of detachment and protecting them from biological degradation. However, few attempts have been reported to combine these strategies in MEAs for electrochemical sensing.

Although ultra-flexible MEAs demonstrate stable neural recording, the success of these devices for long-term electrochemical sensing *in vivo* needs further investigation. Host tissue responses such as protein fouling, inflammatory cell attachment, and scarring exert additional challenges to analyte detection because biofouling interferes with electrochemical detection [138,157,158]. Additionally, tissue damage around the electrode hinders neurotransmitter diffusion to the electrode surface, distorting measurements [74]. A new flexible sensing MEA has been developed and functionalized for multisite monitoring of spinal GLU signaling during myocardial ischemia and reperfusion in a large-animal model [51]. GluOx was crosslinked with bovine serum albumin (BSA) and glutaraldehyde on three of the six Pt electrode sites of the MEA (140 µm wide and ~16 µm thick, 6 × 100-µm diameter electrodes), while the remaining three were used as sentinel sites. An m-Phenylenediamine (mPD) film served as a size-exclusion screening layer to prevent electroactive species other than H_2_O_2_ from reaching the Pt electrode [51].

Probe flexibility offers significant advantages in terms of its capability to move with the neural tissue during micro-motion that might be caused by cardiac pulsation or respiration [51]. Unlike stiff probes, the flexible design minimizes tissue damage and electrode breakage caused by large movement displacements. It also reduces motion artifacts, ensuring more reliable and robust recordings without causing significant trauma in the neural tissue. This is the first report of a flexible GLU sensor used in a large-animal model, highlighting the flexible probe’s potential to advance neural monitoring in preclinical disease models [51].

**Figure 4:**
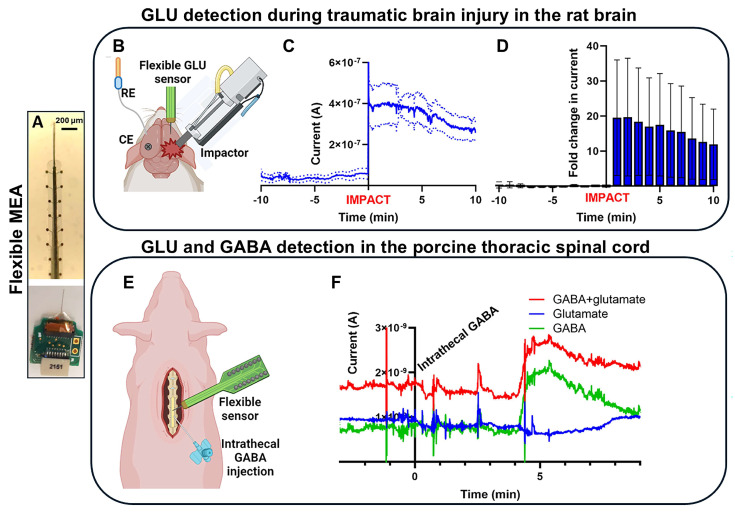
(**A**) An optical image of an assembled flexible MEA with a sharpened tungsten wire (**top**) and the MEA inserted into the ZIF connector of the custom PCB (**bottom**). (**B**–**D**) GLU detection during traumatic brain injury (TBI) in the rat brain. (**B**) A schematic illustration of the setup used to conduct this experiment, including the reference electrode (Ag/AgCl wire), a counter electrode (bone screw), a flexible GLU sensor (enzymatic functionalized MEAs), and the impactor used to create the injury. (**C**) The effect of TBI on the GLU baseline, showing a drastic increase immediately after the impact, and (**D**) remained elevated at over 10 times the baseline level for 10 min post-impact. (**E**,**F**) Simultaneous GLU and GABA detection in the porcine thoracic spinal cord using enzymatic functionalized flexible MEA. (**E**) A schematic illustration of the experimental procedure for measuring GLU and GABA in the porcine thoracic spinal cord. A flexible MEA, functionalized for GLU and GABA detection, was inserted into the thoracic spinal cord, with GABA injected near the implantation site. (**F**) Simultaneous detection of GLU and GABA in the pig spinal cord. GABA is administered (t = 0) through a catheter a few millimeters from the MEA. Adapted from [50]. Copyright © 2024 The Authors. Published by American Chemical Society. This publication is licensed under CC-BY 4.0.

In a later study, nanoPt-coated MEAs, enzymatically functionalized for GLU detection, were developed to improve sensitivity, stability, and footprint (Figure 3A–D). The flexible MEAs were custom-designed with electrode sites spaced 200 μm apart to prevent channel crosstalk. During enzyme drop casting, meticulous care was taken to keep the coating confined to the electrode sites [50]. Modifying platinum microelectrodes with nanometer-scale roughness significantly enhances sensor sensitivity and extends enzyme stability to three weeks [50]. The increased surface area provides more anchor points for GluOx, reducing enzyme loss over time. It also improves H_2_O_2_ oxidation during enzymatic reactions, enhancing electrode sensitivity (Figure 3D). *In vivo* tests in rodent brains showed that nanoPt GLU sensors remained functional for up to seven days, while smooth electrodes failed by day three. Additionally, increasing the effective surface area without enlarging the geometric footprint enables the design of high-density MEAs with a small cross-section [50]. These flexible nanoPt-coated MEAs (Figure 4A) effectively monitored GLU concentration changes in a rat TBI model (Figure 4B–D) [50]. Baseline GLU levels were recorded for 10 min before TBI was induced using an impactor (Figure 4C,D), resulting in a drastic GLU increase, sustained for at least 10 min post-impact (Figure 4D), consistent with TBI-related changes [50]. This was the first study to achieve continuous GLU sensing throughout cortical impact in the same animal, made possible by the MEA’s ultra-flexible thin-film polymer substrate, which adapted to tissue displacement. Additionally, similarly prepared flexible nanoPt-coated MEAs were successfully used to detect both GABA and GLU in the pig spinal cord (Figure 4E,F) [50].

Table 2 summarizes MEAs, both rigid and flexible, functionalized with biological recognition elements that have been used for *in vivo* detection of single or multiple analytes, with or without electrophysiological recordings.

The promising results achieved with implantable flexible devices highlight the transformative potential of flexible and ultra-flexible MEAs, not only in reducing tissue damage and maintaining sensor functionality, but also in overcoming challenges such as tissue displacement and motion artifacts in electrochemical sensing. Despite their promise, only a few examples of flexible MEAs have been reported.

Nanostructured coatings such as platinum nanoparticles and Pt/rGO nanoparticles have shown interesting results in reducing impedance and enhancing sensor sensitivity by increasing surface area [50,131,149,150], which provides additional anchor points for the enzyme and improves stability over time [50]. Despite these advancements, very few studies have reported *in vivo* recordings over multiple days or weeks, highlighting a need for further exploration in long-term applications.

Alternatively, aptamers—ssDNA, RNA, XNA strands artificially synthesized and selected to have high binding affinity to target molecules—can be immobilized on the microelectrode surface [44,53,54,55,56].

**Figure 5:**
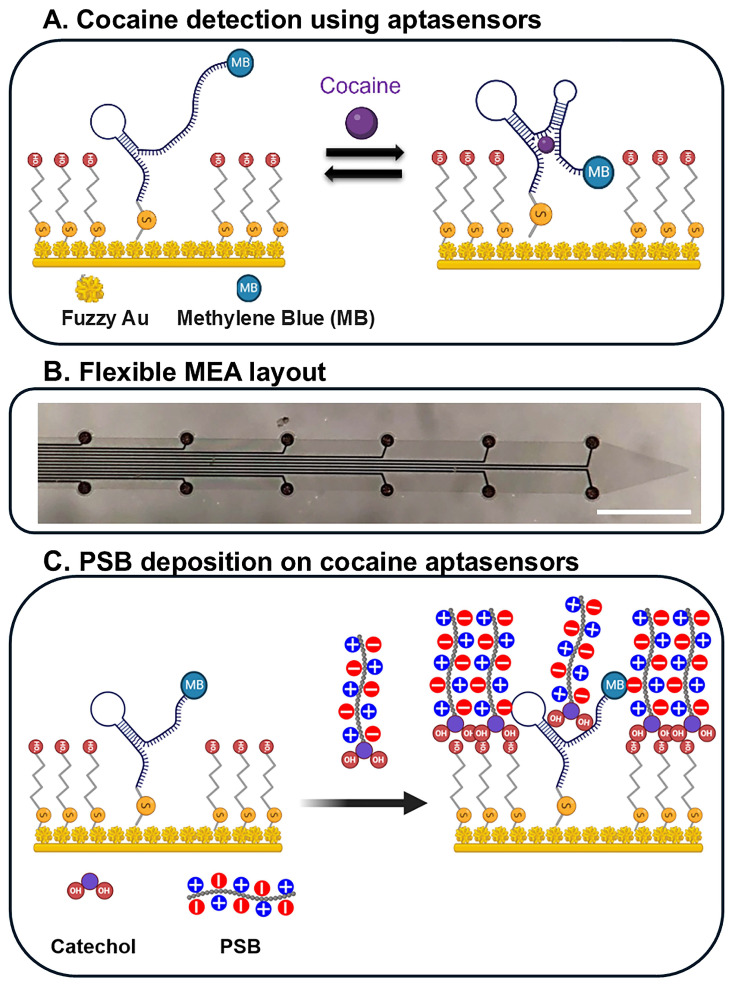
(**A**) Schematic illustration of the assembly process for aptasensors and their detection mechanism. Initially, fuzzy Au was electrodeposited onto the microelectrodes. Aptamers were then immobilized on the fuzzy Au surface, followed by the addition of a self-assembled monolayer of 6-mercapto-1-hexanol (MCH) as a passivation layer. When cocaine binds to the aptamer, a conformational change brings the methylene blue (MB) tag closer to the surface, increasing the current response. (**B**) A flexible MEA featuring 12 gold electrode sites (35 µm in diameter) spaced 300 µm apart along both edges of the shank. Scale bar: 300 µm. (**C**) A schematic depiction of zwitterionic poly(sulfobetaine methacrylate) (PSB) deposition on cocaine aptasensors. The fabricated MEAs, after aptamer functionalization, were immersed in a 10 mM tris buffer containing 2 mg/mL of PSB polymer for 2 h, allowing the coating to be deposited. The PSB polymers were anchored to the sensor surface via their catechol functional groups, bonding to the hydroxyl groups. Adapted from [44]. Copyright © 2023 by the authors. Licensee MDPI, Basel, Switzerland. This article is an open access article distributed under the terms and conditions of the Creative Commons Attribution (CC BY) license.

When target molecules bind to aptamers, they cause reversible conformational change that encloses the molecule within the aptamer’s structure. This reversible binding guarantees that the target molecule is neither consumed nor absorbed in the process [160]. Electrochemical aptamer-based sensors have become a promising method for detecting biomolecules [160,161]. In aptasensors, aptamers can be used in combination with field-effect transistors (FETs) to monitor changes in surface charge [162,163,164,165,166] or they can be functionalized with electrochemical reporter molecules, including methylene blue (MB), ferrocene, or anthraquinone, thus enabling direct detection of analyte binding through electrochemical methods [44,159,167,168,169,170]. A schematic illustration of the assembly process for an aptasensor and its detection mechanism is reported in Figure 5A.

A wide range of aptamers have been developed for different target molecules, including cocaine [44,159,171], ATP [172,173,174], DA [145,175,176], and 5-HT [177,178,179]. However, only a few aptamer-functionalized MEAs have been successful for *in vivo* biochemical sensing [44,159]. For example, the incorporation of cocaine aptamers into implantable silicon MEAs enabled effective cocaine detection and electrophysiological recordings in rat brains. However, the sensor’s signal degraded within 1 h of implantation [159]. Histological analysis identified a layer of biological material, including plasma proteins and microglial cells, forming on the electrode sites, potentially impairing the sensor’s performance. To minimize undesired brain tissue responses and enhance stability in *in vivo* applications, an aptasensor was constructed on a flexible MEA with an SU-8 substrate [44] (Figure 5B).

Beyond mechanical considerations, surface chemistry plays a crucial role in the initial interactions between the device and host tissue. Since 90% of tissue is water, a promising antifouling approach involves coating the surface with a nanometer-thick layer of a zwitterionic polymer, creating a super-hydrophilic interface. This hydration layer effectively prevented protein adsorption and cell attachment [180,181]. Consequently, zwitterionic poly(sulfobetaine methacrylate) (PSB) coatings were applied as a non-fouling protective layer on these flexible aptamer-based MEA sensors to protect the implanted sensors from biofouling and to protect aptamers from enzymatic degradation in the extracellular environment. A schematic representation of the PSB deposition on cocaine aptasensors is reported and described in Figure 5C. The efficacy of the PSB coating in maintaining sensor stability was evaluated both in vitro and *in vivo* [44]. The PSB coating protected the sensors from albumin fouling and DNase-1 enzyme degradation. *In vivo* studies demonstrated that PSB-coated MEA aptasensors could detect repeated cocaine infusions in the brain for up to 3 h post-implantation without any loss in sensitivity [44]. Furthermore, the same MEAs could record electrophysiological signals from different tissue depths simultaneously. This innovative flexible MEA, integrated with cocaine sensors, provides a valuable tool for studying the mechanisms of cocaine addiction. Additionally, the PSB coating technology offers a generalizable solution to enhance the performance of implantable devices by mitigating biofouling and inflammatory host responses [44].

While aptamer sensors offer high selectivity for target molecules, a major limitation *in vivo* is their rapid degradation by nucleases, particularly for RNA-based aptamers, which are highly susceptible to enzymatic breakdown in biological fluids. This breakdown results in a short half-life, restricting their applications to acute studies.

## 5. Integration of Carbon Material in Flexible MEAs

In the previous section, we discussed the advantageous strategy of coating metal MEAs with PEDOT-nanocarbon, where the incorporation of nanocarbon materials significantly enhances the stability and sensitivity of the coating, enabling the detection of dopamine levels using both square wave voltammetry (SWV) and chronoamperometry, concurrently with electrophysiological recordings. Another strategy is the direct integration of carbon into the flexible substrate, which eliminates the need for a coating layer, thereby avoiding potential issues such as degradation and delamination. Additionally, carbon materials offer a much higher electrochemical potential window and electrochemical stability compared to PEDOT, making this integration particularly beneficial for FSCV that requires scanning to high voltages and very fast scan rates.

Carbon is considered the ideal material for electrochemical detection of electroactive neurotransmitters due to its biocompatibility, sensitivity, capacitive electrochemical behavior, wide potential electrochemical window, fast electron transfer kinetics for neurochemical redox reactions, and excellent electrochemical stability [45,47,48,109,136,137,182,183,184]. Carbon fiber microelectrodes (CFEs), used in combination with FSCV, are considered the gold standard for measuring rapid neurotransmitter changes due to their small size (5–10 µm), biocompatibility, flexibility, and favorable electrochemical properties. However, traditionally assembled CFEs are typically limited to a single electrode site and manually encased in borosilicate glass [125,185,186]. These configurations are inserted into the brain using micromanipulators and guide cannulas, which can cause substantial tissue damage, posing challenges for chronic studies [185,186]. Recent advancements have introduced single CFEs [187,188] and CFE arrays [189,190,191,192,193,194,195] insulated with a poly(p-xylylene) or parylene thin-film coatings and designed as subcellular-scale probes for chronic implants. These configurations have enabled longitudinal measurements of sub-second evoked DA fluctuations over one year in rats [188] and for more than 100 days in non-human primates [196]. CFEs coated with conductive polymer coatings at the recording site have demonstrated stable results in chronic *in vivo* recordings, significantly reducing immune responses [187,197]. However, despite their promising capabilities, the fabrication of these electrodes remains highly manual and human-dependent, limiting scalability and feasibility for large-scale, batch production. Wafer-scale batch fabrication is crucial as it enables the simultaneous production of multiple devices with consistent quality and reproducibility that are essential for scaling up advanced technologies. Replacing metal with carbon in the wafer-scale batch fabrication of flexible MEAs, traditionally used for electrophysiological recordings, presents a transformative solution for integrating neurotransmitter detection capabilities. This innovation enables the realization of stable multimodal electrochemical and electrophysiological recordings by leveraging the superior electrochemical properties of carbon materials alongside the enhanced biocompatibility of thin-film devices. However, only a few groups have successfully achieved batch fabrication of carbon-based MEAs on flexible substrates. The primary challenge is the high temperatures required for carbon synthesis, which are incompatible with polymeric substrates. The solution to this challenge, the transfer of pre-patterned carbon structures from silicon wafers onto thin, flexible substrates, raises additional challenges [136,198,199].

**Figure 6:**
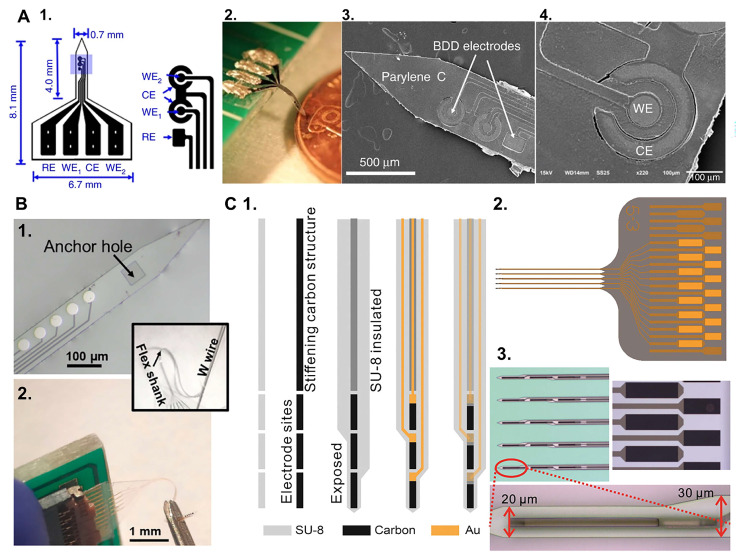
(**A**) Prototypes of a 2-channel boron-doped polycrystalline diamond (BDD) probe for electrochemical sensing and neural recording. (1.) Layout of the BDD probe. (2.,3.) Optical and SEM images of the implantable BDD probe. (4.) SEM close-up view of the BDD working electrode (WE) and counter electrode (CE). Adapted from Fan et al. [198]. Copyright © 2020, Springer Nature. Distributed under the terms of the Creative Commons CC BY. (**B**) Flexible GC-coated hybrid MEA (GC microelectrodes and metal interconnection). (1.) Optical picture of the hybrid GC-MEA on SU-8 flexible substrate. In the inset: different view of the flexible MEA shank featuring an anchor hole at the tip, allowing for the insertion of a 50 µm tungsten shuttle to aid in probe handling and brain penetration. (2.) Flexible hybrid GC-MEAs connected to the PCB using a zero-insertion force (ZIF) connector. Adapted from Castagnola et al. [43] Copyright © 2022 by the authors. Licensee MDPI, Basel, Switzerland. This article is an open access article distributed under the terms and conditions of the Creative Commons Attribution (CC BY) license. (**C**) Design and fabrication of GC fiber-like microelectrode arrays (GCF MEAs). (1.) The fabrication process of the GCF. SU-8 is spin-coated and patterned on a wafer, followed by pyrolysis to produce carbon electrode sites and the stiffening structure. SU-8 is spin-coated and patterned to insulate the stiffening structure while exposing the electrode sites. Metal is patterned using lift-off procedures. The top layer of SU-8 is patterned to insulate the metal patterns. (2.) The layout of the GCF array. (3.) Optical microscopy images of the GCF array. Adapted from Castagnola et al. [106]. Copyright © 1999–2025 John Wiley & Sons, Inc. Distributed under the terms of the CC BY 4.0.

An example of MEAs that use carbon microelectrodes on flexible substrates is a diamond-based microelectrode probe consisting of multichannel boron-doped polycrystalline diamond (BDD) microelectrodes integrated onto a soft Parylene C substrate [198] (Figure 6A). The fabrication process involved growing microcrystalline BDD films on wafers using microwave plasma-assisted chemical vapor deposition (MW-PACVD). These films were then patterned into microelectrodes using an aluminum mask in an electron cyclotron resonance reactive ion etcher (RIE). The pre-patterned BDD structures were subsequently transferred onto Parylene C films, with the growth side exposed as the sensing electrodes [198]. Morphological and electrochemical performance evaluations have demonstrated that electrodes fabricated from the BDD growth side exhibit superior characteristics compared to those made from the nucleation side. Specifically, the growth surface electrodes display a rougher morphology, higher sp^3^ content, wider water electrochemical potential window, and faster dynamic kinetics. The nanoscale roughness and large grain size of the BDD microelectrodes increase their effective surface area, thereby reducing electrochemical impedance and minimizing noise during electrophysiological recordings, both in vitro and *in vivo* [198]. Additionally, electrodes fabricated from the BDD growth surface demonstrate improved in vitro DA sensitivity and a lower tendency for biofouling [198]. However, despite these advantages, diamond requires doping to achieve acceptable conductivity and does not achieve comparable sensitivity to smaller carbon electrodes, such as carbon CFEs [46,63] or GC [43,200].

Another example of MEAs with carbon microelectrodes on flexible substrates is the GC-MEA, which are obtained by integrating GC microelectrodes into flexible devices with metal interconnects, using a pattern transfer fabrication technique developed by Dr. Kassegne’s lab [135,199,201]. GC microelectrodes are synthesized through the pyrolysis of a pre-patterned polymeric SU-8 precursor in an inert, controlled atmosphere. The possibility to photolithographically pre-pattern the SU-8 as a negative photoresist eliminates the need for post-patterning steps. The resulting GC microelectrodes are then transferred onto a flexible polymeric substrate [134,135,201,202]. The ability to precisely control the pyrolysis conditions plays a crucial role in achieving high-quality GC electrodes, which are essential for the sensitive and reliable performance of the GC-MEAs. This technique has been successfully used to incorporate GC microelectrodes into 40 µm thick, polyimide-insulated implantable MEAs [200,202,203,204], which can penetrate brain tissue without external aid. These implantable MEAs, with GC microelectrodes and metal interconnections (hybrid GC-MEAs), demonstrated high sensitivity for FSCV detection of DA and 5-HT in vitro, with good resistance to electrochemical fouling [200]. They also achieved high quality acute single-unit and local field potential recordings in rat [203] and songbird [202] cortices. When implanted in the songbird striatum, they enabled both *in vivo* detection of DA and high-quality single-unit recordings [204].

Recent advancements have enabled the miniaturization of these hybrid GC-MEAs on thin, flexible SU-8 substrates (Figure 6B) [43,107]. These flexible GC-MEAs achieved multisite electrochemical recordings of DA and 5-HT, and they exhibited reduced tissue damage and inflammation compared to stiff silicon probes, preserving a healthier neural tissue interface [107]. However, their flexibility necessitates a metal wire shuttle for penetration into deeper brain regions and they have a larger footprint than single CFEs [43,107].

To enable self-insertion, the fabrication method has been adapted for batch production of GC fiber-like (GCF) MEAs [106] (Figure 6C). This novel design incorporates fiber-like GC structures on thin-film substrates, with an additional GC-fiber-like backbone that stiffens the MEAs for insertion but remains disconnected from the metal interconnections. The long, relatively stiff GCF structures, with an extremely small cross-section, allow the device to penetrate the brain tissue without aid. GCF MEAs also exhibit enhanced sensitivity toward DA and 5-HT compared to 7 μm CFEs. GCF MEAs have been successfully used to measure tonic DA and 5-HT concentrations *in vivo* when combined with optimized SWV waveforms and to detect stimulation-evoked phasic DA via FSCV, from the same implanted device. They also recorded single-unit electrophysiological activity in the striatum of mouse brains. This novel design holds significant promise for multimodal measurements of neural activity and neurotransmitter concentrations while maintaining an exceptionally minimal footprint [106].

**Figure 7:**
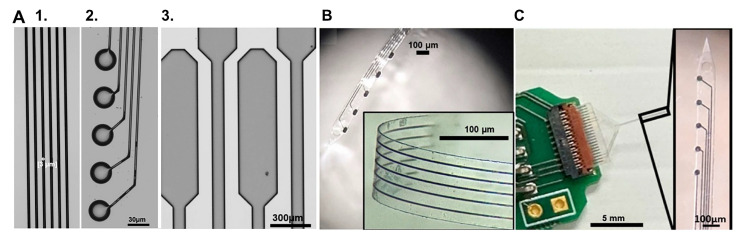
“all” GC-MEAs with CG electrodes and interconnects. (**A**) 1. GC traces, 2. GC microelectrodes, and 3. GC connection pads after patterning and carbonization on silicon substrate. (**B**) “all” GC-MEAs during the peeling off from the PDMS with a zoomed-in view of the folding in the inset. Shank size: 140 µm wide and ~8 µm thick. (**C**) Insulated “all” GC-MEAs connected to the custom-made printed circuit board (PCB) using a zero-insertion force (ZIF) connector. The inset shows a magnification of the corresponding shank (120 µm wide and ~8 µm thick, with 30 µm diameter circular microelectrodes). Adapted from Faul et al. [205]. Copyright © 2024 by the authors. Licensee MDPI, Basel, Switzerland. This article is an open access article distributed under the terms and conditions of the Creative Commons Attribution (CC BY) license.

Another fabrication technique, named the double-pattern transfer photolithographic process, has been developed for the fabrication of flexible polyimide GC-MEAs with GC electrodes and interconnects (“all” GC-MEAs) [136], to address potential concerns regarding the adhesion between metal interconnections and GC electrodes (hybrid GC-MEAs), which may not withstand prolonged and aggressive electrical or mechanical stresses in chronic applications. The first “all GC” prototype demonstrated exceptional performance, including in vitro FSCV DA detection and robust electrochemical stability during prolonged current delivery [136]. These results highlight their potential for creating stable neural interfaces. However, further miniaturization of these “all GC” designs is required to achieve chronic electrochemical sensing applications. To advance this effort, Dr. Castagnola’s lab recently published a proof-of-concept study on the fabrication of GC MEAs with GC electrodes and interconnections on thin, flexible substrates with significantly reduced dimensions [205] (Figure 7). The study explored the strengths and limitations of two microfabrication methods: (1) a double pattern-transfer photolithographic process, that transfer-bonds the MEAs on a temporary polymeric support, and (2) a double-etching process, that uses a 2 µm-thick low-stress low-pressure chemical vapor deposition (LPCVD) nitride (Si_3_N_4_) coating of the Si wafer as the bottom insulator layer for the MEAs, eliminating the need for pattern-transfer. Although both methods demonstrated feasibility, further optimization is needed to achieve the process control and scalability required for reliable batch fabrication, particularly for devices with miniaturized features [205].

An alternative approach to fabricating devices with carbon electrodes and interconnections is the laser-induced carbonization process on polymeric substrates. Laser-induced graphene (LIG), directly grown from commercially available polyimide (PI) or tailored PIs via laser irradiation, has gained attention for the synthesis of large-area, porous graphene materials with tunable pore density, ideal for advanced electrode designs [206,207,208,209,210,211]. This approach enables the direct writing of graphene features on flexible substrates with facile patterning and high throughput production. The porous morphology, atomic structure, and chemical composition of LIG are controllable by tuning the laser parameters such as laser power, raster speed, laser spot size, and spot overlap [206,212]. Additionally, molecular control of PI composition can be used effectively to tailor heteroatom compositions of the resulting LIG without the need for external doping sources [207].

A novel graphene-based neural interface, named “NeuroString”, has been recently developed to seamlessly integrate with the central nervous system (CNS) and gastrointestinal (GI) tissue using an LIG method [213]. Transition metal nanoparticles, specifically 5,10,15,20-tetrakis-(4′-aminophenyl) iron (III) porphyrin chloride, and 5,10,15,20-tetrakis-(4′-aminophenyl) nickel (II) porphyrin, were incorporated into a polyamic acid polymer precursor to produce nanoparticle-modified graphene networks via a CO_2_ LIG process [213]. The resulting nanoporous graphene network demonstrated exceptional electrochemical properties. Combined with the advantages of rapid laser patterning and an efficient transfer process enabled by the polystyrene block-poly(ethylene-ran-butylene)-block-polystyrene (SEBS) polymer as an insulator, this innovative platform enables the rapid fabrication of customized patterns. 3-channel NeuroString sensors (90 × 50 μm^2^ in size) were temporarily rigidified using pullulan coating to assist the implantation in the mouse brain, and they were successfully used for multi-channel FSCV detection of optically stimulated and behavioral DA dynamics in mouse the nucleus accumbens (NAc), and optically stimulated the 5-HT response in the basolateral amygdala (BLA) [213]. Chronic implantation studies demonstrated that NeuroString provides reproducible DA signals for up to 16 weeks, along with enhanced biocompatibility and reduced tissue response compared to conventional rigid probes [213].

Although LIG technology offers numerous advantages, including rapid prototyping and material versatility, it is constrained by the resolution limits of the laser’s spot size [214]. This limitation affects the fabrication of extremely fine features, particularly those smaller than 50-100 µm, which are essential for advanced miniaturized devices. The typical line widths achieved with LIG (50–100 µm) [213] are significantly larger than a single CFE (7 µm) [124], or GC traces, which can be fabricated down to 3 µm [205].

While the integration of carbon materials into flexible, batch-fabricated devices has enabled reliable multimodal sensing, minimized tissue damage, and improved long-term stability, continued advancements in carbon microelectrode technologies, such as novel fabrication techniques and miniaturization, are crucial for advancing chronic neurochemical sensing and enhancing multimodal neural interface designs. Despite these promising advancements, the fabrication of carbon electrodes on flexible substrates remains more complex and challenging than the standard fabrication processes used for flexible metal MEAs. Thus, while PEDOT composite coatings on metal electrodes may be easier to achieve, carbon electrodes continue to offer significant advantages due to their superior long-term stability and wide electrochemical window, particularly when using FSCV. The superior stability and versatility of carbon make it an attractive choice for applications requiring sustained performance over time.

Table 3 summarizes flexible microelectrode arrays (MEAs) with integrated carbon electrodes, as well as carbon electrodes and interconnections on flexible polymeric substrates.

## 6. Acquiring Electrochemical and Electrophysiological Measurements from a Single Device

To comprehensively understand the dynamic interactions between chemical and electrical communication in neuronal circuits, integrated tools capable of simultaneously measuring chemical release (electrochemistry) and electrical activity (electrophysiology) within the central nervous system (CNS) are indispensable. To achieve dual measurement, it is essential to combine MEAs with multimodal capabilities with hardware designed to collect and integrate these datasets without electrical crosstalk. A detailed review of the various strategies used to achieve combined electrochemical and electrophysiological measures can be found elsewhere [81].

In summary, two main approaches have been used and are summarized below.

The *parallel approach* involves the use of separate microelectrodes dedicated to either electrochemical or electrophysiological acquisition. For example, different ceramic MEAs, with sites dedicated to either one or the other measurements, have been developed and used for multi-channel amperometry detection of neurotransmitters and electrophysiological (LFP/spike activity) acquisition [82,131,144,149,150,215,216]. The two datasets are collected from spatially separate sites. However, the inter-site distance is known, and inter-experiment variability is limited by the reproducibility of MEA manufacturing techniques. Using this approach, simultaneous multi-analyte detection and electrophysiological activity recording have also been recently achieved [91,132,149]. Chronoamperometry and constant potential amperometry measurements did not significantly impact neuronal activity *in vivo*, with only brief crosstalk occurring at the beginning and end of the recording. These transient electrical artifacts lasted for 3 to 5 ms [82,215].

The *serial or sequential approach* uses a single microelectrode to measure both neurochemical releases, and single-unit neuronal activity by alternating between recording modes [217,218]. This serial approach involves switch circuits that allow “time sharing” between recording modes, and has been used for the detection of DA [218] and/or oxygen [219,220] by FSCV while recording single-unit neural activity at the same microelectrode in the interval between FSCV scans. This is possible thanks to the sub-second temporal resolutions of the FSCV that align with the timescale of chemical dynamics at neuronal synapses [95,221,222,223,224]. During these combined experiments, a triangular waveform is applied at 5 Hz, half the normal FSCV frequency. Every scan takes less than 10 ms and scans are repeated at 200 ms, to provide ~180 ms of electrophysiological recording between scans [225,226]. The advantage of this strategy is that both datasets are recorded at the same microelectrode, thus the same cell population/brain region is studied. Descriptions of the hardware used for combined electrochemical and electrophysiological recordings in awake animals are summarized in [227,228]. This approach has only been performed with one recording site at a time, but it can be extended to MEAs.

For the multimodal measurements described in this paper, involving SWV in combination with FSCV and/or electrophysiology, SWV measurements were performed sequentially on the same recording sites due to instrument limitations [39,43,106]. This sequential scanning introduced a temporal lag across different channels, requiring the sensing data to be averaged over 5 min time bins, which underutilized the temporal resolution of the technique. A multichannel potentiostat system that can simultaneously scan all channels will eliminate this issue [39].

An alternative approach, fast sampling amperometry (FSA), has been recently investigated [81]. This approach utilizes high-frequency constant-potential amperometry for seamless single-sensor recordings of neurochemical and electrophysiological events. At a 40 Hz sampling rate, a four-site MEA captured LFPs and electrochemical signals, linking acetylcholine release to hippocampal theta oscillations [229]. With higher sampling rates (100–1000 Hz), this method demonstrated concurrent recordings of metabolic and oxygen fluctuations with LFPs in rodent models [81,230]. This approach allows for real concurrent recordings in both the temporal and spatial domains, requiring a single instrument [81].

For all the approaches mentioned, a significant limitation lies in the limitations of hardware used to conduct multichannel simultaneous electrochemical measurements.

The novel MEA technologies enable the concurrent recording of neural electrical signals and neurotransmitter detection, providing a comprehensive platform to explore the complex interactions between electrical and chemical signaling in the brain. Designed with flexible polymers and functionalized surfaces, these MEAs have shown great promise in reducing mechanical trauma and inflammation, supporting stable, long-term multimodal neural recordings.

One key development is the use of nanomaterials, particularly nanocarbon-based conductive polymer composites, which enhance the electrochemical performance of the MEAs. These materials offer a larger surface area, low-impedance electrodes that improve the signal-to-noise ratio in electrophysiological recordings, and increased sensitivity and stability for extended neurotransmitter monitoring.

Another transformative development is the substitution of metals with carbon in the batch fabrication of flexible MEAs. This innovation improves both electrochemical performance and biocompatibility, facilitating stable, multimodal electrochemical and electrophysiological recordings through the advantageous properties of carbon materials.

Neurotransmitter-selective electrochemical detection, which relies on the immobilization of biological recognition elements such as enzymes or aptamers on the microelectrode’s surface, has also been substantially improved through the use of nanoporous surfaces, flexible MEA substrates, and zwitterionic polymer encapsulation. However, enzyme and aptamer degradation in biological environments remains a challenge, and current immobilization methods present limitations. For example, manual drop casting provides low throughput and can result in variable coating coverage and thickness. Crosslinking may also negatively affect the electrode impedance for electrophysiology. Improved immobilization techniques are needed to improve coating precision, increase throughput, and enhance device stability and performance, particularly for long-term monitoring in complex biological environments.

As these MEA technologies continue to evolve, their application in understanding the complex interactions between electrical and chemical signaling in the brain will provide valuable insights into neurological disorders and the effects of therapeutic interventions. To fully realize the potential of these MEA devices for multimodal measurements, specialized hardware is needed to efficiently collect and integrate electrochemical and electrophysiological datasets without causing electrical interference.

Current approaches for multimodal acquisition are limited by either spatial or temporal resolution, as well as channel count, reducing both efficiency and precision in multimodal mapping of the brain. These constraints emphasize the need for improved hardware to enable high channel count, high-resolution, and simultaneous measurements. Advancements in miniaturization, data processing, and integration are essential to overcoming these challenges. Such improvements will allow for more effective management of complex datasets, expanding the utility of flexible MEAs in both research and clinical settings. Ultimately, these developments will lay the foundation for next-generation diagnostic tools and therapies, significantly advancing the field of neuroscience.

## 7. Conclusions and Future Directions

This review highlights recent advancements in flexible MEAs designed for both electrophysiological and electrochemical monitoring of neural activity. These developments include strategies to integrate neurotransmitter detection at multiple time scales (tonic and phasic), enable simultaneous detection of multiple analytes, and employ methods to minimize sensor fouling and brain damage for long-term performance.

Novel MEA technologies enable the concurrent recording of neural electrical signals and neurotransmitter detection, providing a comprehensive platform to explore the complex interactions between electrical and chemical signaling in the brain. Designed with flexible polymers and functionalized surfaces, these MEAs have shown great promise in reducing mechanical trauma and inflammation, supporting stable, long-term multimodal neural recordings.

One key development is the use of nanomaterials, particularly nanocarbon-based conductive polymer composites, which enhance the electrochemical performance of the MEAs. These materials offer a larger surface area, low-impedance electrodes that improve the signal-to-noise ratio in electrophysiological recordings, and increased sensitivity and stability for extended neurotransmitter monitoring.

Another transformative development is the substitution of metals with carbon in the batch fabrication of flexible MEAs. This innovation improves both electrochemical performance and biocompatibility, facilitating stable, multimodal electrochemical and electrophysiological recordings through the advantageous properties of carbon materials. However, the batch fabrication of carbon electrodes on flexible substrates remains more complex and challenging than the standard fabrication processes used for flexible metal MEAs. Therefore, continued advancements in carbon microelectrode technologies, including novel fabrication techniques and miniaturization, are essential for advancing chronic neurochemical sensing and improving multimodal neural interface designs.

Neurotransmitter-selective electrochemical detection, which relies on the immobilization of biological recognition elements such as enzymes or aptamers on the microelectrode’s surface, has also been substantially improved through the use of nanoporous surfaces, flexible MEA substrates, and zwitterionic polymer encapsulation. However, enzyme and aptamer degradation in biological environments remains a challenge, and current immobilization methods present limitations. For example, manual drop casting provides low throughput and can result in variable coating coverage and thickness. Crosslinking may also negatively affect the electrode impedance for electrophysiology. Improved immobilization techniques are needed to improve coating precision, increase throughput, and enhance device stability and performance, particularly for long-term monitoring in complex biological environments.

As these MEA technologies continue to evolve, their application in understanding the complex interactions between electrical and chemical signaling in the brain will provide a deeper understanding of the mechanisms underlying neurological disorders, one of the leading causes of disabilities worldwide. This progress will have profound implications for the treatment of neurological and brain disorders, offering diagnostic value and supporting advanced therapies such as closed-loop deep brain stimulation, other neuromodulation-based therapy, and therapeutic brain–machine interfaces (BMIs) [231,232,233,234,235,236].

To fully realize the potential of these MEA devices for multimodal measurements, specialized hardware is needed to efficiently collect and integrate electrochemical and electrophysiological datasets without causing electrical interference.

Current approaches for multimodal acquisition are limited by either spatial or temporal resolution, as well as channel count, reducing both efficiency and precision in multimodal mapping of the brain. These constraints emphasize the need for improved hardware to enable high channel count, high-resolution, and simultaneous measurements. Advancements in miniaturization, data processing, and integration are essential to overcoming these challenges. Such improvements will allow for more effective management of complex datasets, expanding the utility of flexible MEAs in both research and clinical settings. Ultimately, these developments will lay the foundation for next-generation diagnostic tools and therapies, significantly advancing the field of neuroscience.

Additionally, success toward multimodal reading of brain activities may open new avenues for deeper understanding of the basis for intelligence and even consciousness, enabling engineers to simulate its activities, advancing the ability to reverse-engineer the brain. For example, a better understanding of brain neural dynamics can inspire the development of sophisticated artificial neural networks and AI algorithms, enhancing performance in tasks like pattern recognition and decision-making. Unlike today’s computers, which rely on binary logic gates, replicating the brain’s variable levels of neuronal excitation could lead to the development of far more powerful and efficient computing machines.

In addition to their use in electrophysiology and electrochemical sensing, PEDOT-based coatings and carbon microelectrodes can also be employed for electrical stimulation [119,212,237,238,239,240,241,242]. Furthermore, recent advances in ultra-flexible devices have enabled stable chronic intracranial macrostimulation with low currents, ensuring seamless tissue integration and avoiding neural degeneration. These advances suggest that tissue-integrated electrodes can provide effective, selective, and long-lasting neuromodulation while minimizing tissue damage and off-target effects [243].

While the dual sensing modality, including multi-analyte detection, provides significant insights into brain mechanisms, stimulation is particularly valuable for applications such as neuromodulation therapies [231,232,233], the brain–machine interface [244,245,246], and the promotion of tissue regeneration [247,248]. The integration of stimulation capability on these devices not only expands their functionality but also enables more comprehensive and targeted treatments for neurological disorders and enhanced neural plasticity.

A promising future direction is the integration of flexible MEAs with organoid models, offering significant advantages for studying brain activity and testing therapies [249,250]. Notably, pluripotent stem cell-derived brain organoids have made remarkable progress [251,252,253,254,255], replicating key features of the human brain with their 3D multicellular architecture and developmental profile.

Flexible MEAs would be particularly beneficial due to their ability to conform to the organoid’s 3D structure, creating a biomimetic environment for more relevant electrical and mechanical stimulation. Unlike rigid MEAs, they enhance electrophysiological recordings by adapting to soft, curved surfaces while also accommodating organoid growth and structural changes for non-invasive monitoring. This combination will advance research on diseases such as neurodegenerative and development disorders and cancer.

## Figures and Tables

**Table 1:** MEAs functionalized with conductive polymers and composite coatings for electrophysiological recording and neurotransmitter detection ^1,2^.

Device	Coating	Modality	Sensitivity	LOD	Echem Technique	Acute/Chronic	Ref.
CFEs and silicon MEAs	PEDOT/CNT	Echem (tonic DA)	108 ± 9 (CFEs) and 14.7 ± 0.05 nA/μM (MEAs) in a CSF	2.03 ± 0.09 nM (CFEs)	SWV	Acute (rat brain)	[124]
Flexible GC-MEAs	PEDOT/CNT	Echem (tonic and phasic DA)	55.634 ± 0.001 nA/μM in aCSF	--	SWV (at PEDOT/CNT) and SWV (at GC)	Chronic SVW (21 days) and acute FSCV (mouse brain)	[43]
Flexible GC-MEAs	PEDOT/CNT	Echem (tonic 5-HT)	17.56 ± 0.01 nA/μM	--	SWV	Acute and chronic (7 days) (mouse brain)	[107]
Silicon MEAs	SWCNTs/PEDOT:PSS	Ephys and Echem (DA)	217 pA/μM	10 nM	chronoamperometry	Acute (rat brain)	[130]
Flexible MEAs	PtNPs/PEDOT:PSS	Ephys and Echem (DA)	162.3 pA/μM in PBS	--	chronoamperometry	Acute (mouse brain)	[132]
Flexible MEAs	PEDOT/CNT	Ephys and Echem (tonic DA)	~150 nA/μM in PBS	~4.4 nM	SWV	Chronic (28 days) (mouse brain)	[39]
Flexible MEAs	rGO/PEDOT:PSS	Ephys and Echem (DA)	15 pA/μM in PBS with agarose	--	CPA	Chronic (6 weeks)(mouse brain)	[133]

^1^ Abbreviations: artificial cerebrospinal fluid (aCSF), carbon fiber microelectrodes (CFEs), electrophysiology (Ephys), electrochemistry (Echem), dopamine (DA), serotonin (5-HT), microelectrode arrays (MEAs), phosphate-buffered saline (PBS), square wave voltammetry (SWV), glassy carbon (GC), poly(3,4-ethylenedioxythiophene) (PEDOT)/carbon nanotubes (CNT)-coated (PEDOT/CNT), rGO/PEDOT:PSS, platinum black nanoparticles and PEDOT:PSS (PtNPs/PEDOT:PSS), reduced graphene oxide (rGO)-doped PEDOT:PSS (rGO/PEDOT:PSS), single-wall carbon nanotube (SWCNTs/PEDOT:PSS). ^2^ The sensitivity is reported as reported in the original papers.

**Table 2:** MEAs functionalized with biological recognition elements for electrophysiological recording and neurotransmitter detection ^1,2^.

Device	Electrode Material	Modality	Biological Recognition Elements	Sensitivity	Echem Technique	Acute/Chronic	Ref.
Silicon MEAs	Pt/rGOs nanoparticles	Ephys and Echem (DA and GLU)	GluOx and mPD on the GLU site	8.62 ± 1.32 pA/μM for GLU 13.21 ± 2.31 pA/μM for DA (in PBS)	chronoamperometry	Acute (mouse brain)	[131]
Ceramic-based MEAs	Pt	Echem (GLU and ACh tonic and phasic)	GluOx and mPD (GLU) ChOx/AChE (ACh)	4.2 ± 2.0 pA/μM (GLU) 5.8 ± 2.6 pA/μM (ACh)	chronoamperometry	Acute (rat brain)	[89]
Silicon MEAs	Pt	Echem (GLU and GABA)	GluOx and mPD GluOx/GABASE and mPD	~500 nA/μM.cm^2^ (in PBS)	chronoamperometry	Chronic (11 days) (rat brain)	[52]
Flexible MEAs		Echem (GLU and GABA)	GluOx and mPD	--	chronoamperometry	Acute (pig spinal cord)	[51]
Silicon MEAs	nanoPt	Echem (GLU)	GluOx and mPD	1.590 ± 0.057 × 10^−2^ nA/μM (in PBS)	chronoamperometry	Acute and chronic (7 days mouse brain)	[50]
Flexible MEAs	nanoPt	Echem (GLU)	GluOx and mPD	1.590 ± 0.057 × 10^−2^ nA/μM (in PBS)	chronoamperometry	Acute (TBI rat brain)	[50]
Flexible MEAs	nanoPt	Echem (GLU and GABA)	GluOx and mPD GluOx/GABASE and mPD	1.590 ± 0.057 × 10^−2^ nA/μM (in PBS)	chronoamperometry	Acute (pig spinal cord)	[50]
Bimodal (RTBM) microelectromechanical system (MEMS) neural prob	Pt coated with mPD and OPPy	Ephys and Echem (glucose, lactate, GLU, and choline)	glucose oxidase, LOx, ChOx, GluOx, and mPD	6.18 ± 0.71 nA mM^−1^ (glucose) 0.62 ± 0.07 nA mM^−1^ (lactate) 7.03 ± 1.26 pA μM^−1^ (GLU) and 19.82 ± 1.09 pA μM^−1^ (choline) (in aCSF)	chronoamperometry	Acute (mouse brain)	[148]
Silicon MEAs	PtNPt	Ephys and Echem (GLU)	GluOx and mPD	7.807 pA/μM (in PBS)	chronoamperometry	Acute (rat brain)	[149]
Silicon MEAs	PtNPt	Ephys and Echem (GLU)	GluOx and mPD	56 pA µM^−1^ (in PBS)	chronoamperometry	Acute (rat brain)	[150]
Silicon MEAs	dendritic gold	Ephys and Echem (cocaine)	Cocaine-targeting aptamer: 5′-HS-(CH2)6-AGACAAGGAAAATCCTTCAATGAAGTGGGTCG-(CH2)7-MB-3′ and MB	Not linear modified exponential Langmuir model	SWV	Acute (rat brain)	[159]
Silicon MEAs	fuzzy gold	Ephys and Echem (cocaine)	Cocaine-targeting aptamer: 5′-HS-(CH2)6-AGACAAGGAAAATCCTTCAATGAAGTGGGTCG-(CH2)7-MB-3′ and MB	Not linear modified exponential Langmuir model	SWV	Acute (rat brain)	[44]

^1^ Abbreviations: artificial cerebrospinal fluid (aCSF), acetylcholinesterase (AChE), choline oxidase (ChOx), electrophysiology (Ephys); electrochemistry (Echem), glutamate oxidase (GluOx), glutamete (GLU), lactate oxidase (Lox), methylene blue (MB), reduced graphene oxide nanocomposites (Pt/rGOs), phenylenediamine (mPD), Platinum nanoparticles (PtNPt or nanoPt), phosphate-buffered saline (PBS), square wave voltammetry (SWV), overoxidized polypyrrole (OPPy), γ-aminobutyric acid (GABA). ^2^ The sensitivity is reported as reported in the original papers.

**Table 3:** Flexible MEAs with integrated carbon electrodes, carbon electrode, and interconnections on flexible polymeric substrate ^1,2^.

Device	Electrode Material	Modality	Sensitivity	LOD	Echem Technique	Acute/Chronic	Ref.
FlexibleMEAs	BDD	Ephys and Echem (DA)	0.9 nA/µM (in PBS)	830 nM	SWV	Acute ephys in rat	[198]
hybrid GC-MEAs	GC	Echem (DA and 5-HT)	164 nA/µM (DA) and 110 nA/µM (5-HT) using EW −0.4/1 V at 400V/s 354 nA/µM (DA) and 170 nA/µM (5-HT) using EW −0.5/1.3 V at 400 V/s. (in PBS)	1.11nM (DA) and 1.29 nM (5-HT) using EW −0.4/1 V at 400 V/s. 1.17 nM (DA) and 1.73 nM (5-HT) using EW 0.5/1.3 V at 400 V/s	FSCV	Acute (proof of concept) co-detection of DA and 5-HT in the rat striatum	[200]
hybrid GC-MEAs	GC	Echem (DA)	105.18 ± 6.22 nA/µM (in aCSF)	--	Multichannel FSCV	Acute mouse DS	[43]
hybrid GC-MEAs	GC	Ephys and Echem (DA)	--	--	FSCV	Acute, songbird striatum	[204]
GCF MEAs	GC	Ephys and Echem (DA and 5-HT)	FSCV: 2.0 ± 0.2 pA µM^−1^ µm^−2^ (DA) and 4.0 ± 0.2 pA µM^−1^ µm^−2^ (5-HT). SWV: 0.45 µA cm^−2^ nM^−1^ (DA) and 1.22 µA cm^−2^ nM^−1^ (5-HT) (in PBS)	FSCV: 1.18 (DA) 0.89 nM (5-HT)	FSCV and SWV	Acute, mouse and rat brain	[106]
“all” GC-MEAs	GC	Ephys and Echem (DA)	1.135 nA/nM.cm^2^ (in PBS)	10nM	FSCV	Ephys from rats	[136]
“all” GC-MEAs	GC	Echem (5-HT)	122.94 ± 4.36 nA/μM (in PBS)	--	FSCV	--	[205]
Neuro String	graphene/Fe_3_O_4_ nanoparticle network embedded in an elastomer	Echem (5-HT and DA)	--	--	FSCV	Chronic detection mouse brain (DA) and acute detection mouse brain (5-HT) and mouse colon and pig gut	[213]

^1^ Abbreviations: artificial cerebrospinal fluid (aCSF), boron-doped polycrystalline diamond (BDD), dopamine (DA), dorsal striatum (DS), electrophysiology (Ephys), electrochemistry (Echem), electrochemical window (EW), fast scan cyclic voltammetry (FSCV), glassy carbon (GC), GC Microelectrode arrays with GC microelectrode and metal (“all” GC-MEAs) interconnections (hybrid GC-MEAs), GC Microelectrode arrays with GC microelectrode and interconnections (“all” GC-MEAs), phosphate-buffered saline (PBS), square wave voltammetry (SWV), serotonin (5-HT). ^2^ The sensitivity is reported as reported in the original papers.

## Abbreviations

The following abbreviations are used in this manuscript:

MEAs	Microelectrode arrays
DA	Dopamine
5-HT	Serotonin
AD	Adenosine
ACh	Acetylcholine
MT	Melatonin
GLU	Glutamate
GABA	γ-aminobutyric acid
H2O2	Hydrogen Peroxide
XNA	Xeno Nucleic Acid
FSCV	Fast Scan Cyclic Voltammetry
SWV	Square Wave Voltammetry
CFEs	Carbon Fiber Microelectrodes
GN	Guanosine
M-ENK	Methionine-enkephalin
CPA	Constant potential amperometry
m-PD	m-phenylenediamine
AA	ascorbic acid
DOPAC	3,4-dihydroxyphenylacetic acid
CNT	carbon nanotubes
GC	Glassy carbon
FSCAV	fast-scan controlled-adsorption voltammetry
GCF	GC fiber-like
PPy	Polypyrrole
GO	graphene oxide
SWCNTs	single-wall carbon nanotube
NSR	Signal-to-noise-ratio
WT	wild type
MU	Δ19 Clock mutant
rGO	Reduced graphene oxide
PtNPs	platinum nanoparticles
nanoPt	Nano Platinum
TBI	traumatic brain injury
FETs	field-effect transistors
MB	methylene blue
PSB	poly(sulfobetaine methacrylate)
BDD	polycrystalline diamond
MW-PACVD	microwave plasma-assisted chemical vapor deposition
RIE	reactive ion etcher
LPCVD	low-stress low-pressure chemical vapor deposition
LIG	Laser-induced graphene
NAc	Nucleus Accumbens
BLA	Basolateral Amygdala
LFPs	Local Field Potentials
CNS	Central Nervous System
FSA	Fast Sampling Amperometry
PEDOT/CNT	poly(3,4-ethylenedioxythiophene) (PEDOT)/carbon nanotubes (CNT)-coated (PEDOT/CNT)
